# Supplementary material for: Novel Bacteriophage Specific against Staphylococcus epidermidis and with Antibiofilm Activity
Source: Viruses. 2022 Jun 20;14(6):1340. doi: 10.3390/v14061340 (PMC9230115; doi:10.3390/v14061340)
Supplement: Supplementary file 1 [file viruses-14-01340-s001.zip › viruses-1733067-supplementary.pdf]

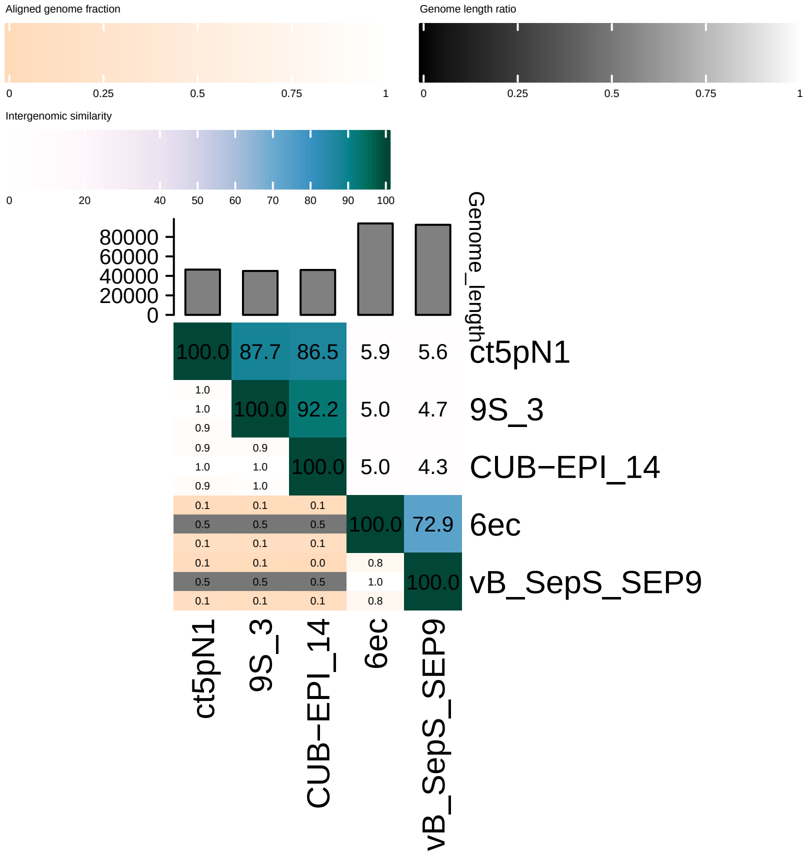

Aligned genome fraction
0
0.25
0.5
0.75
1
Genome length ratio
0
0.25
0.5
0.75
1
Intergenomic similarity
0
20
40
50
60
70
80
90
100
80000
60000
40000
20000
0
Genome_length
100.0
87.7
86.5
5.9
5.6
ct5pN1
1.0
1.0
0.9
100.0
92.2
5.0
4.7
9S_3
0.9
1.0
0.9
0.9
1.0
1.0
100.0
5.0
4.3
CUB−EPI_14
0.1
0.5
0.1
0.1
0.5
0.1
0.1
0.5
0.1
100.0
72.9
6ec
0.1
0.5
0.1
0.1
0.5
0.1
0.0
0.5
0.1
0.8
1.0
0.8
100.0
vB_SepS_SEP9
ct5pN1
9S_3
CUB−EPI_14
6ec
vB_SepS_SEP9
